# Monte Carlo approach to fuzzy AHP risk analysis in renewable energy construction projects

**DOI:** 10.1371/journal.pone.0215943

**Published:** 2019-06-13

**Authors:** Luis Serrano-Gomez, Jose Ignacio Munoz-Hernandez

**Affiliations:** Departamento de Mecánica Aplicada e Ingeniería de Proyectos, Universidad de Castilla-La Mancha, Albacete, Castilla-La Mancha, España; Indian Institute of Technology Madras, INDIA

## Abstract

The construction of large renewable energy projects is characterized by the great uncertainties associated with their administrative complexity and their constructive characteristics. For proper management, it is necessary to undertake a thorough project risk assessment prior to construction. The work presented in this paper is based on a hierarchical risk structure identified by a group of experts, from which a Probabilistic Fuzzy Sets with Analysis Hierarchy Process (PFSAHP) was applied. This probabilistic analysis approach used expert opinion based on the Monte Carlo Method that allows for extracting more information from the original data. In addition, the coherence of the experts’ opinions is assessed using a novel parameter known as Confidence Level, which allows for adjusting the opinions of experts and weighting their judgments regarding impact and probability according to their coherence. This model has the advantage of offering a risk analysis in the early stages of the management of renewable energy projects in which there is no detailed information. This model is also more accurate than the classic fuzzy methodology when working with complete distribution functions, whilst it avoids the loss of information that results from the traditional mathematical operations with Fuzzy numbers. To test the model, it was applied to a 250 MW photovoltaic solar plant construction project located in southeast of Spain (Region of Murcia). As a result of the application of the proposed method, risk rankings are obtained with respect to the cost, the time, the scope and from a general point of view of the project.

## Introduction

In some countries, the reduction or disappearance of economic incentives to produce from renewable energy sources has caused a drastic decline in the investment in new projects. However, the maturity of renewable technologies has led to a fall in the costs of the main technological components. This has, in turn, led to a resurgence of the photovoltaic sector, in which activity is focused on the large plants, which, even at current market prices, provide sufficient profitability due to economies of scale. It is important to bear in mind that undertaking a large project entails managing budgets to the value of tens of millions of euros, in addition to considerable administrative complexity. Such large projects are often processed at the level of Central Governments and National Transport Network Operators, which entails slower and more laborious procedures compared with those of local and regional organizations. If a construction project of a large photovoltaic plant is developed without conducting an analysis of the possible risks of the project from an early stage, this could incur considerable losses for the promoter of the company. Various definitions of risk have been proposed in the literature, although perhaps more appropriate for renewable projects is that adopted by Perry and Hayes [1] and Chapman and Ward [2]. These authors regard risk as ‘exposure to the possibility of economic or financial loss or gain, physical damage or injury, or delay, as a consequence of the uncertainty associated with the pursuing a particular course of action'.

Risk assessment and risk management is a topic that has been widely described and studied in Aven [3], where definitions and metrics of risks are analyzed, as well as how to deal with issues such as uncertainty, robustness and resilience. This work focuses on both qualitative and quantitative risk analysis, based on prior risk identification [4]. To deal with the vagueness of qualitative data in the early phase of a project, this work develops a risk analysis methodology based on the fuzzy sets theory. The fuzzy sets theory [5] establishes a definition of the data using simple language terms such as lower risk, high probability, or moderate impact. From the linguistic terms, and using fuzzy logic, it is possible to implement a mathematical method that allows for the development of the risk analysis process, by removing the subjectivities.

There are several studies on risk assessment using fuzzy sets. For instance, Li and Wang [6] established a risk assessment methodology based on fuzzy AHP for PPP projects in China, whilst Alizera et al. [7] studied risk probability and risk impact on highway construction projects in Iran using fuzzy ANP. Ferreira and Franklin [8] used fuzzy sets for risk assessments in nuclear systems; Xiaojun et al. [9] applies fuzzy sets for green product development; Yao-Chen and Shih-Tong [10] employed consistent fuzzy preference relations in metropolitan construction projects; and Nieto-Morote and Ruz-Vila [11] applied fuzzy sets theory to risk assessment in the rehabilitation project of the Polytechnic University of Cartagena. Studies employing fuzzy logic can also be found in renewable energy research. For instance, Garcet and Volg [12] analyzed the risk policy for prioritizing renewable energy projects; Shafiee [13] used fuzzy ANP to analyze marine wind park operation risks; Chen [14] used fuzzy logic for risk assessment in wind farm construction projects; Liu et al. [15] studied risk analysis in photovoltaics integration building projects in China; Jiuping et al. [16] applied fuzzy logic in an attempt to minimize costs, deadlines, blockage, and environmental impact in a hydroelectric construction project; and Yan et al. [17] identified the biomass gasification system using bow-tie analysis, identifying the causes and consequences of gas leakage.

A combination of fuzzy sets and probabilistic analysis with Monte Carlo simulation has also been employed in numerous studies, such as that of Yu et al. [18] in which both of these methods were used to predict peak power and to plan power systems. Wu et al. [19] used fuzzy sets to study the likelihood of wind, and Monte Carlo simulation to predict the thermal energy produced by a wind turbine, and Içen and Demirham [20] studied the appropriate error measures for the estimation of fuzzy linear regression model parameters using the Monte Carlo method. According to the literature review, the fuzzy AHP is an applicable approach for multi-attribute decision-making process [21], and has been used to carry out risk assessments under conditions of uncertainty. Using this method, it is also possible to generate the weights and priorities of risks from the pair-wise comparison matrices of expert judgments [22]. Although the weight of expert opinion is an important parameter to consider and has been widely used in previous work [23], it is insufficient when a risk assessment method is posed in which, in addition to comparing the influence of risks at each of the hierarchy levels, the probability and impact of the risks in the project framework are assessed individually.

To overcome the limitations of the fuzzy AHP technique, this paper presents a methodology for the qualitative and quantitative analysis of risks, which differs principally from other fuzzy methods of risk analysis. The chief contributions of this work are: 1) it introduces a new parameter known as Confidence level, which allows for variations in the opinions of experts according to their coherence and weighting their judgements regarding impact and probability, 2) it employs a probabilistic analysis of the opinion functions instead of applying the mathematical simplifications used in other methods of fuzzy analysis, which allows for extracting more information from the same data.

This approach provides a smooth application with a rapid and thorough methodology that could be readily used for both qualitative and quantitative risk analyses based on linguistic assessments in the early stages of large-scale renewable construction projects. A risk ranking is then generated, which subsequently forms the basis of a risk response strategy. This paper describes the proposed methodology, providing a step-by-step explanation of how the model can be developed and applied to any type of building project. Finally, the model is applied to a large real-life photovoltaic solar plant project located in Spain, and the results yielded from this field application are then discussed.

## Research method

Classic Fuzzy methods for risk analysis share the same general structure [24]:

1. Parameter definition and measurement: this defines the basic parameters, risk probability, and risk impact. The parameter measurement is conducted using linguistic terms and these are converted into fuzzy numbers. If the group of experts has an extensive knowledge of the sector, then they can issue concise opinions using linguistic terms, which subsequently become triangular numbers [25].
2. Fuzzy inference definition: the relationship between the input and output parameters can be defined as relations "if-then" or in the form of mathematical functions defined by an appropriate fuzzy arithmetic operator.
3. Defuzzification: as result of the previous phase, a fuzzy number is obtained, which, at this phase is represented by an exact numerical value.

In the current work, the identified risks are organized according to a hierarchical structure, which allows them to be prioritized based on four fundamental factors: project risk impact, risk probability, risk discrimination (always defined in linguistic terms) and the risk factor function, defined as a distribution function.

As a progression of the classic method, pair-wise risk comparisons are introduced [7, 11, 26, 27, 28, 29]. On some occasions, experts must give their opinions on areas where their expertise is minimal, and it is therefore necessary to evaluate the consistency of their judgments. This evaluation of consistency has also been referred to in many studies. Bin et al. [30], for instance, used the eigen value method to determine the consistency of the matrices, and Monte Carlo simulation to optimize its consistency, whilst Shafiee [13] used the Buckley method.

This work presents a risk assessment model that introduces a parameter to evaluate the consistency of expert judgments, allowing for the weighting of opinions regarding risk probability and impact, as well as calculating the weighting coefficients of the experts’ discrimination of the risks based on the consistency of their judgments from transitivity laws.

Once the risk functions have been added within each group and incorporated into the risk structure, the final phase uses the proposed method to obtain a ranking of general risk influence on the project framework. A detailed risk analysis allows for an evaluation of the influence of risk on scope, time and/or cost of the project.

One of the main drawbacks of the classical fuzzy sets theory concerns the use of mathematical operations with fuzzy numbers, since operating with triangular or trapezoidal numbers results in a triangular or trapezoidal number, respectively. @Risk tool is used for probabilistic analysis using Monte Carlo simulation to achieve greater precision in mathematical operations, which allows for working directly with the triangular fuzzy numbers distribution functions.

S1 Fig shows a three-phase model based on probabilistic analysis and the fuzzy sets theory. The phases are: risk definition phase, measurement and weighting phase, and fuzzy inference phase.

### Risk definition phase

#### Establish an expert group for risk identification

The selected experts must have a high degree of knowledge and previous experience in similar renewable energy projects [31].

The ethics committee that approved the study is the "Comité de Ética de Investigación con Medicamentos" of the Human and Health Care Service of the Government of the Region of Castilla-La Mancha. The internal code is 2018/11/129.

The experts were selected from professionals who have collaborated on the processing, design, and construction of photovoltaic solar plants, with the aim of covering as many profiles as possible involved in the development of such plants. A total of 15 experts were recruited with the following profiles:

E1: Developer of photovoltaic solar plants, with a 15 MW portfolio of completed projects
E2: Project Manager, with a portfolio of designed and developed projects exceeding 500 MW worldwide.
E3: CEO of a multinational company with 14 years of experience in the sector and more than 2 GW of developed projects.
E4: Construction Manager of solar photovoltaic plants, with more than 10 years of experience in the sector.
E5: O & M Manager of a multinational company with a portfolio of over 2 GW.
E6: Project Finance manager in a multinational company, with more than 14 years of experience in the promotion and development of photovoltaic solar plants.
E7: Supervisor in the construction of renewable energy projects—both wind and photovoltaic—with 12 years of experience in the sector.
E8: Project Manager, with over 300 MW of developed projects worldwide.
E9: Industrial Engineer and expert in design and construction of energy evacuation infrastructures in photovoltaic solar plants, with more than 30 years experience in the design of electrical infrastructures.
E10: Inspector-Verifier of electrical installations, with 4 years of experience in the sector.
E11: Professional Project Manager and expert in the construction of large electric power generation infrastructures.
E12: Project Manager, with a portfolio of projects exceeding 50 MW, designed and constructed in Spain.
E13: Forestry Engineer and Public Administration technician responsible for the environmental evaluation of projects.
E14: Project Manager, with a portfolio of projects exceeding 20 MW, designed and constructed in Spain.
E15: Construction Manager of solar photovoltaic plants and solar thermal plants, with more than 12 years of experience in the sector.

All the experts confirmed their acceptance and gave their verbal consent to complete the questionnaire. Experts E4, E8 and E14 declined to complete the questionnaire due to lack of time. The other 12 experts are those included in the present study.

#### Risk identification and construction of a risk breakdown structure

The risk assessment group is needed to identify and classify the project risks. Tools to facilitate the identification of risks include: Checklist, Influence Diagrams, Cause and Effect Diagrams, Failure Mode and Effect Analysis, Hazard and Operability Study, Fault Trees, and Event Tree [32, 33, 34].

Based on a general classification of the project risks, such as Political Risks, Technical Risks, Economic Risks, Time Delay Risks, Legal Risks and Social Risks, the risk identification and hierarchical risk structures are constructed in two phases.

In the first phase, the experts were asked to respond to the following question: "Based on the general classification of risks, identify the possible general conditions that may affect the project for each of them". We then obtained the second level of the hierarchical risks structure i.e. the risks subgroups.

The second phase focused on the identification of specific risks that arise in the project. The question that the experts were asked in this phase is, "For each of the risks subgroups that form the hierarchical structure, please identify and describe the risks that may arise in the project”.

As a result, a hierarchical risk structure is generated to organize risks in suitable detail, enabling further evaluation to proceed efficiently [30, 35].

### Measuring and weighting phase

#### Defining the overall risk factor function

A definition of the overall risk factor function is usually based on two fundamental factors in risk assessment: Risk Impact (RI) and Risk Probability (RP).

Based on the axiom established in Kaplan and Garrick [36] in relation to the comparability of the uncertainty, for a complete risk assessment, a third parameter must be considered, known as Risk Discrimination (RD) [37].

The risk discrimination parameter is based on the pair-wise risk comparison at the different levels that make up the hierarchical structure.

For each evaluated risk, a risk factor function value is defined using Eq (1):

$$ORF\;=\;\frac{RI}{RDI}\times \frac{RP}{RDP}$$ (1)

where *ORF* is the overall risk factor function, *RI* is risk impact, *RP* is risk probability, *RDI* is risk discrimination impact, and *RDP* is risk discrimination probability in the project life cycle.

### Defining the linguistic scale and fuzzy numbers

In projects with great uncertainty, it is difficult to numerically assess the risks due to a lack of accurate information. In these cases, the judgments of the group of experts are collected through linguistic expressions.

According to the fuzzy sets [11, 38, 39], in a universal set X, a fuzzy subset A of X is defined by a membership function μ_A_ (x), which assigns each element x in X to a real number in the interval [0, 1].

Having defined the linguistic terms used, to proceed with their analysis, they must be transformed into fuzzy numbers using an appropriate conversion scale that considers both the concept and the context of the expressions.

According to the research carried out by Zeng et al [26], the triangular function is used for the identified risk analysis. For this purpose, and with the aim of being able to carry out a subsequent analysis to determine the coherence of the experts’ opinions, scales of five terms were established to determine both the impact and probability of the risks. In addition, a scale of nine terms, symmetrical with respect to the fifth value, was used for the pair-wise comparison between risks. In this way, the opinions of the experts are transversally comparable.

The linguistic terms used, their meaning, and the triangular function assigned are shown in S2–S4 Figs.

#### Sending and collecting questionnaires: RI, RP, and RD measures

To gather the expert judgements, questionnaires were sent out by email directly to the addresses provided by the experts.

Having established the hierarchical risks structure, the questionnaire was organized as follows:

1. General information on the project.
2. Step-by-step instructions.
3. Experts’ opinions about risk probability.
4. Experts’ opinions about the impact of risk on the project scope.
5. Experts’ opinions about the impact of risk on the project costs.
6. Experts’ opinions about impact of risk on project development/execution time.

Using these questionnaires, the experts can give their opinions using the linguistic terms indicated in S2–S4 Figs. The column matrix Pgm is generated from the questionnaires, from RP opinions with respect to the general project framework (RPim), as shown in Eq (2),

Pgm=[RP1mRP2m…RPnm] (2)

where *i* is the risk identification number at the lower hierarchy level, *P* is the *RP* matrix of the group *g*, for expert *m*.

To conduct a detailed risk analysis, *RI* opinions are examined on the influence of the risks at Cost (*RI*_*Ci*_^*m*^), at Time (*RI*_*Ti*_^*m*^) and at the project Scope (*RI*_*Si*_^*m*^), generating Impact column matrix *I*_*Cg*_^*m*^, *I*_*Sg*_^*m*^, *I*_*Tg*_^*m*^, according to Eq (3),

ICgm=[RIC1mRIC2m…RICnm];ISgm=[RIS1mRIS2m…RISnm];ITgm=[RIT1mRIT2m…RITnm] (3)

where *I* is the *RI* matrix over the Cost (*C*), the Scope (*S*) or the Time (*T*) of the group *g*, for the expert *m* and *i* is the risk identification number.

Regarding the opinions about parameter *RD*, the panel of experts provided their comparative judgements between each pair of risks for each one of the hierarchy levels, as well as assessing their probability, along with their impact on cost, time, or the scope of the project. The result is the *nxn* discrimination matrix defined in Eq (4),

$$E_{gl}^{m}=\left[\begin{array}{cccc} - & \left(RD_{12}^{m}\right) & \ldots & \left(RD_{1n}^{m}\right)\\ \left(RD_{21}^{m}\right) & - & \ldots & \left(RD_{2n}^{m}\right)\\ \ldots & \ldots & \ldots & \ldots \\ \left(RD_{n1}^{m}\right) & \left(RD_{n2}^{m}\right) & \ldots & - \end{array}\right]$$ (4)

where *E* is the *RD* matrix, *n* is the number of risks that make up the group *g* at the level *l* of the hierarchy, and *m* is the expert identification number in the risk assessment group.

#### Confidence level

When experts provide an opinion about *RI* or *RP*, they do so individually for each risk, but they should be consistent with their judgements on the *RD* pair-wise comparison.

To measure the consistency of experts’ opinions between *RI* and *RDI*, and between *RP* and *RDP*, a new factor defined as the Confidence Level (*CL*) is introduced.

The linguistic scales for the opinions on *Ri* and *Rp* established in S2 and S3 Figs are scales of 5 terms or levels. The First Level corresponds to negligible impact or very low probability, and the Fifth Level corresponds to critical impact or very high probability.

The Linguistic scale for the Opinions about *Rd* Set out in S4 Fig is a scale of nine Symmetrical terms with respect to the central term, "Same". The difference between the opinion established by the expert with the central term of the scale is defined as a step, so that steps can be obtained from value 0 to ± 4. Thus, it is established that an expert m will be fully in line with their opinions if the absolute difference in levels between the experts’ opinions about *RI* or *RP* for a risk *i* and *j* at the same hierarchical risks structure sub-group, is equal to the absolute value of the steps in *RD*, according to Eqs (5) and (6).

$${CL(RI)}_{i}^{m}=ABS(ABS({LRI}_{i}^{m}-{LRI}_{j}^{m})-ABS({DSI}_{ij}^{m}))$$ (5)

$${CL(RP)}_{i}^{m}=ABS(ABS({LRP}_{i}^{m}-{LRP}_{j}^{m})-ABS({DSP}_{ij}^{m}))$$ (6)

When an expert gives a judgment that is not 100% coherent, this incoherence allows for the qualification and weighting of their opinions. To qualify an expert’s opinion, the opinion dispersion module (*DM*) is previously determined as the ratio between the minimum interval *T*, defined as the interval between the likely triangular fuzzy numbers values at the opinion scale, and the product between the minimum *CL* admitted as valid and the maximum number of discriminations between risks in the same group, defined as the *n* risk number which comprises the largest group *g*, less 1.

$$DM\;=\;\frac{T_{min}}{{CL}_{min}\times (n_{g}-1)}$$ (7)

Then, the adjusted RI* or RP* opinion for an expert *m* at the *i* risk is,

$${RI}_{i}^{m*}={RI}_{i}^{m}+(({LRI}_{i}^{m}-{LRI}_{j}^{m})-{DSI}_{ij}^{m})\times DM$$ (8)

$${RP}_{i}^{m*}={RP}_{i}^{m}+(({LRP}_{i}^{m}-{LRP}_{j}^{m})-{DSP}_{ij}^{m})\times DM$$ (9)

As an example, if an expert considers that the impact of risk A is Serious (4) and the impact of Risk B in the same group is Minor (2), the levels difference is 2. If the expert is consistent in his discrimination judgements, then Risk A generates a Bit More impact (+ 1) than Risk B (- 1) on the general project framework, and their RD absolute difference is 1, so applying Eq (5), the CL is 75%.

Thus, the opinion regarding Risk A is adjusted to the left (- 1xDM) of its original position (Serious), and the opinion of Risk B is adjusted to the right (- 1xDM) of its original position (Minor), as shown in S5 Fig.

From the Confidence Level the quality of both the risk definition and description is assessed. To do this, a minimum coherence threshold is set in such a way that if the *CL* is lower than the minimum coherence threshold for at least half of the experts, the risk definition and description is not correct, since it is not logical to suppose that half or more of the experts are not coherent in their risk opinions.

In this case, the risk definition and description must be modified, and the experts should again give their opinions on *RP*, *RI*, and *RD* in all groups and hierarchy levels associated with the redefined risk.

## Aggregating an individual fuzzy number into a group: Impact and probability

For those experts where *CL* is greater than the minimum threshold set, the opinions coefficient is estimated by the weighting of *RI* and *RP* experts using Eqs (10) and (11),

$${WRI}_{i}^{m}=\frac{{CL(RI)}_{i}^{m}}{\sum_{n=1}^{m}{CL(RI)}_{i}^{n}}$$ (10)

$${WRP}_{i}^{m}=\frac{{CL(RP)}_{i}^{m}}{\sum_{n=1}^{m}{CL(RP)}_{i}^{n}}$$ (11)

where *WRI*_*i*_^*m*^ and *WRP*_*i*_^*m*^ are the opinions weighting coefficients of *RI* or *RP*, *CL (RI)* and *CL (RP)* are the Confidence Level calculated with Eqs (5) and (6), respectively, for each risk *i* and expert *m*.

In the case that an expert *CL* is below the minimum threshold established, its weighting coefficient will be zero.

Aggregation of the experts’ adjusted opinions is carried out according to Eqs (12) and (13),

$${RI}_{i}=\sum_{n=1}^{m}\frac{1}{{WRI}_{i}^{n}}\times {RI}_{i}^{n}=(\frac{1}{{WRI}_{i}^{1}}\times {RI}_{i}^{1}+\frac{1}{{WRI}_{i}^{2}}\times {RI}_{i}^{2}+\cdots +\frac{1}{{WRI}_{i}^{n}}\times {RI}_{i}^{m})$$ (12)

$${RP}_{i}=\sum_{n=1}^{m}\frac{1}{{WRP}_{i}^{n}}\times {RP}_{i}^{n}=(\frac{1}{{WRP}_{i}^{1}}\times {RP}_{i}^{1}+\frac{1}{{WRP}_{i}^{2}}\times {RP}_{i}^{2}+\cdots +\frac{1}{{WRP}_{i}^{n}}\times {RP}_{i}^{m})$$ (13)

where *i* is each one of the risks in the bottom hierarchy level, and *m* is the expert identification number in the risk assessment group.

### Measuring the RD parameter

Recently, various authors have used pair-wise comparison in their research [6, 9, 30, 35, 40].

In the present work, the evaluation group members discriminate between pairs of risks—in terms of both impact and probability—in the same *g* group for each one of the *l* levels at the hierarchical structure.

Linguistic expressions collected through questionnaires are converted into their corresponding triangular fuzzy numbers, as shown in S4 Fig. For each one of the experts, the *E* comparison matrix is obtained for each *g* group at *l* level in the hierarchy according to Eq (4).

Transitivity [41, 42, 43] is the generally accepted property for dealing with the consistency of fuzzy preference relations. It represents the idea that the preference value obtained by direct comparison of two alternatives would be equal to or greater than the preference value obtained by using an indirect chain.

Application of the transitivity laws is carried out for each one of the possible combinations, calculated according to the following expression,

$$C_{m,n}\;=\;\frac{m!}{m!\times (m!-n!)}$$ (14)

where *m* are the risk numbers in the group and *n* is the comparison order, whose value in the case of transitivity laws is 3. For groups with two risks, it is assumed that experts will not show inconsistency in their opinions.

For the purposes of this work, Weak Transitivity is considered the minimum consistency threshold that must be reached. If an expert’s opinion does not satisfy the Weak Transitivity criterion, their opinion will be eliminated. In turn, additive transitivity is the optimal point to achieve. For each one of the possible combinations, where a value is obtained that is closer to *3xs*_*0*,_ then the more symmetrical the experts opinions. But there is also an additive transitivity application maximum value, which is defined as maximum interval (*MI*), resulting from the consistent combination of the extreme opinions linguistic scale. If the result of applying the additive transitivity law is greater than *MI*, the expert must revise their discriminatory judgements at all levels in the hierarchical structure affected by the pair-wise risks.

Before adding the opinions of the experts, the values can be weighted from the distance between the result obtained from applying the additive transitivity law and its ideal value *3xs*_*0*_.

The weighting of opinions based on the distance to an ideal value has been applied in previous studies [44, 45].

If the obtained value from applying the additive transitivity law to an m expert over the r_i_ and *r*_*j*_ risks is lower than the *MI*, the expert’s opinion can be weighted according to the distance between that value to the ideal *3xs*_*0*_ by using the following expression:

WRDijm=DWijm∑n=1mDWijn=(MI2-(Max/MinATLij-3×s0)ijm)∑n=1m(MI2-(Max/MinATLij-3×s0)ijn) (15)

where *WRD*_*ij*_^*m*^ is the weight for an *m* expert on the comparison between *r*_*i*_ and *r*_*j*_ risks, *DW*_*ij*_^*m*^ is the distance-weighted regarding the *MI* between the farthest from applying the additive transitivity law, *Max/MinATL*_*ij*_, and ideal value *3xs*_*0*_. If the result of applying the additive transitivity law is greater than *MI*, the expert’s weight in this pair-wise comparison of risks will be zero.

Having fixed the expert’s weights, the fuzzy numbers comparative is aggregated into a fuzzy number according to Eq (16),

$${RD}_{ij}=\sum_{n=1}^{m}\frac{1}{{WRD}_{ij}^{n}}\times {RD}_{ij}^{n}=(\frac{1}{{WRD}_{ij}^{1}}\times {RD}_{ij}^{1}+\frac{1}{{WRD}_{ij}^{2}}\times {RD}_{ij}^{2}+\cdots +\frac{1}{{WRD}_{ij}^{n}}\times {RD}_{ij}^{m})$$ (16)

where *i* and *j* are the risks of the *g* group at the *l* level hierarchy and *m* is the number of experts in the risk assessment group.

To operate mathematically with the risk discrimination, it is necessary to transform the square matrix *E* obtained using Eq (3) into a column matrix using the following expression [9, 46]:

Di=(RDi1×RDi2×…×RDin)1n (17)

Assuming that the *r*_*i*_ risk has over t groups at distinct levels in the hierarchical risk structure, and *D*_*gi*_ is the *D-value* of the *g*^*er*^ top group containing the risk *r*_*i*_ in the hierarchy, the *RD* aggregated value for each *r*_*i*_ risk is calculated according to:

$${RD}_{i}\;=\;D_{i}\times \prod_{g\;=\;1}^{t}D_{gi}$$ (18)

where *i* is the risks in the bottom hierarchy level.

### Fuzzy inference phase

#### Estimating ORF and integration

In the final deduction step, risk analysts turn the aggregate distribution functions of *RI*, *RDI*, *RP*, and *RDP* into a distribution function that represents the risk factor for each *r*_*i*_ risk.

Based on the risk impact analysis on the Scope (*S*), Cost (*C*) and project Time (*T*), this determines the risk factor for the Scope, Cost, and/or on Time for each *r*_*i*_ risk, according to Eq (19):

$${ORF}_{Si}=\frac{{RI}_{Si}}{{RDI}_{Si}}\times \frac{{RP}_{i}}{{RDP}_{I}};\;{ORF}_{Ci}=\frac{{RI}_{Ci}}{{RDI}_{Ci}}\times \frac{{RP}_{i}}{{RDP}_{I}};\;{ORF}_{Ti}=\frac{{RI}_{Ti}}{{RDI}_{Ti}}\times \frac{{RP}_{i}}{{RDP}_{I}}$$ (19)

Eq (20) is used to obtain the overall risk factor, whose result is a distribution function that represents the global risk factor for each identified *r*_*i*_ risk, as shown in S6 Fig.

ORFi=(RISiRDISi×RICiRDICi×RITiRDITi)13×RPiRDPi (20)

#### Risk ranking

The final risk ranking is the result of choosing the characteristic distribution function parameter, obtained upon calculating the global risk factor. Working with distribution functions the ranking is based on well Mean, well Mode, and studying their variations. S7 Fig shows a typical Mode Risk Ranking.

## Case study of a renewable energy construction project

To study and apply the proposed risk assessment methodology, we have chosen as a case study a 250 MW photovoltaic plant project located in the village of Jumilla, in the Region of Murcia (Southeast of Spain). This project is in the early phase of study and initial administrative treatment. Given the plant size and the project budget, initially estimated at 250 million euros, the application of a risk assessment methodology is essential from the very beginning of the project, since it allows for identifying the key risks and setting the risk response strategy. The project area is close to 600 Ha, with 1 million polycrystalline photovoltaic panels over fixed structure, whose energy will be channeled into central power inverters 1MVA.

The power inverters output will rise to 30 KV through 250 transformation stations, being connected with the 250 MVA park transformer sub-station and 132 kV output voltage. Finally, the installation will connect a 12 km, 132 kV evacuation line to the connection point with the REE distribution sub-station.

It is clear from the brief description above that both the technical and administrative complexity of the project is very high, and it occupies an extensive space that has a significant social, economic, administrative, and environmental impact on the surrounding area, thus generating project risks. In addition, many risks can affect its development such as network quality and lack of resources.

### Establishment of the risk assessment group

To apply the proposed methodology, a risk assessment group is established, which is composed of twelve experts with extensive experience in photovoltaic construction projects.

### Identifying sources of risk and building the risk breakdown structure

The risk identification and the risk breakdown structure constitute part of the work conducted by Serrano and Muñoz [4], completed with several methods of risk identification available in the risk literature [27, 47, 48, 49].

### Sending and collecting questionnaires

Having identified (Table 1) and structured the risks associated with the project (S8 Fig), questionnaires were produced in which experts expressed their opinions concerning the impact of the risk on the Scope, Cost, and Time of the project, as well as the probability of each one of the 55 identified risks occurring, as shown in Table 2, according to S2–S4 Figs.

**Table 1:** Third level at RBS: Project risks identified.

RISK LIST	
1.1.1.- Level of political stability	3.4.1.- Costs due to inadequate PV cell selection
1.1.2.- The change in energy policy	3.4.2.- Costs due to inadequate inverter selection
1.2.1.- Approval by the Local Body	3.4.3.- Costs due to lack of consistency in the selection of support panels.
1.2.2.- Obtaining the construction license	3.5.1.- Bank financing
2.1.1.- Technological climate change adequacy	3.5.2.- Changes in power demand
2.1.2.- Flood and storm risks	3.5.3.- Inflation
2.1.3.- Estimation of effective solar radiation	3.5.4.- Changes in energy prices
2.1.4.- Earthworks	4.1.1.- Delays in obtaining administrative approval for the connection infrastructure
2.1.5.- Geotechnical study	4.1.2.- Construction delays of the power connection infrastructure
2.2.1.- New PV solar power systems	4.1.3.- Delays in obtaining PV plant Start-up Act.
2.2.2.- PV cell selection	4.1.4.- Delays in the agreement signature with REE and CNMC
2.2.3.- Inverters selection	4.2.1.- Delays in obtaining the Local Body Approval.
2.2.4.- Selection of support panel structure	4.2.2.- Delays in obtaining approval of the environmental impact.
2.2.5.- Connection to the electric grid	4.2.3.- Delays in obtaining the construction license
2.2.6.- Alternative power generation systems	5.1.1.- Specific legislation changes
3.1.1.- Plant operation cost	5.1.2.- General legislation changes
3.1.2.- Corrective maintenance costs	5.2.1.- Legislative changes in the Administrative Authorization of the power connection infrastructure
3.1.3.- Prevention of maintenance costs	5.2.2.- Legislative changes in the Startup Act permits.
3.1.4.- Performance losses	5.2.3.- Obtaining the electrical registration for production facilities
3.2.1.- Errors in estimating the effective solar radiation energy	5.3.1.- Legislative changes in the Local Body Approval
3.2.2.- Revenue estimation due to the climate change	5.3.2.- Legistaltive changes in the Enviromental Impact Approval.
3.2.3.- Earthworks resources	5.3.3.- Legislative changes in the Construction License
3.2.4.- Flood prevention works	6.1.1.- Theft
3.2.5.- Solution of geotechnical problems	6.1.2.- Vandalism
3.3.1.- Connection to electric grid costs	6.1.3.- Terrorism
3.3.2.- Agreement costs with landowners	6.2.1.- Social consequences resulting from land acquisition
3.3.3.- Possibility of constructing the power connection infrastructure	6.2.2.- Social acceptance
3.3.4.- Construction license	

**Table 2:** *RI* and *RP* expert opinions: Sub-group ‘Plant Location’.

RISK	Risk Impact			Risk Probability
	Scope	Costs	Time	
2.1.1.	Minor (0,1;0,25;0,4)	Moderate (0,3;0,5;0,7)	Negligible (0;0,1;0,2)	Very Low (0;0;0,1)
2.1.2.	Negligible (0;0,1;0,2)	Serious (0,6;0,75;0,9)	Moderate (0,3;0,5;0,7)	High (0,7;0,9;1)
2.1.3.	Serious (0,6;0,75;0,9)	Critical (0,8;0,9;1)	Minor (0,1;0,25;0,4)	Low (0;0,1;0,3)
2.1.4.	Negligible (0;0,1;0,2)	Moderate (0,3;0,5;0,7)	Serious (0,6;0,75;0,9)	Low (0;0,1;0,3)
2.1.5.	Minor (0,1;0,25;0,4)	Serious (0,6;0,75;0,9)	Critical (0,8;0,9;1)	Low (0;0,1;0,3)

Triangular fuzzy numbers are entered into the @Risk tool as triangular distribution functions for further analysis through Monte Carlo simulation [50].

As in the previous case, the experts must also discriminate between risks by means of a pair-wise comparison at the three hierarchy levels, in terms of the impact of risk on the scope, cost, and project time, as well as on the risk probability, as shown in Table 3.

**Table 3:** *RD* expert’s response to the questionnaire regarding impact on Scope: Sub-group ‘Plant Location’.

2.1.	2.1.1.	2.1.2.	2.1.3.	2.1.4.	2.1.5.
2.1.1.	-	More (0,125;0,25;0,375)	Bit less (0,5;0,625;0,75)	More (0,125;0,25;0,375)	Much more (0;0,125;0,25)
2.1.2.	Less (0,625;0,75;0,875)	-	Much less (0,75;0,875;1)	Same (0,375;0,5;0,625)	Bit more (0,25;0,375;0,5)
2.1.3.	Bit more (0,25;0,375;0,5)	Much more (0;0,125;0,25)	-	Much more (0;0,125;0,25)	Very Much more (0;0;0,125)
2.1.4.	Less (0,625;0,75;0,875)	Same (0,375;0,5;0,625)	Much less (0,75;0,875;1)	-	Bit more (0,25;0,375;0,5)
2.1.5.	Much less (0,75;0,875;1)	Bit less (0,5;0,625;0,75)	Very Much less (0,875;1;1)	Bit less (0,5;0,625;0,75)	-

In the literature, there are several studies concerned with discrimination between risks, but these always focus on the impact of the risk on the overall project framework. However, discriminating risks in terms of their impact on Scope, Cost, and Project Time along with their probability is a key contribution that allows for a more extensive and exhaustive risk analysis.

### Data treatment

Once the questionnaires have been compiled, these are introduced in the model, and the Confidence Level is calculated using Eqs (5) and (6). CL allows for an evaluation of the coherence of the opinions given by the experts. In addition to structuring and describing the risks, the opinions are adjusted according to Eqs (8) and (9), weighting their assessment judgements over the risk impact and risk probability of the 55 identified risks using Eqs (10)–(13).

Aggregation of the experts adjusted opinions was carried out as a weighted sum according to Eqs (12) and (13), where the weights are determined from the *CL* according to Eqs (10) and (11).

The aggregated results for Risk 2.1.2 form a normal distribution, as can be seen in Table 4.

**Table 4:** Risk Impact on Scope: Aggregation for ‘Flood and storm risks’.

RISK	EXPERT	RIC Measure	CL (%)	RIC* Measure	WEIGHTS
2.1.2.	E1	(0;0,1;0,2)	75	(0,01;0,11;0,21)	0,111
	E2	(0,3;0,5;0,7)	50	(0,24;0,44;0,64)	0,074
	E3	(0,6;0,75;0,9)	75	(0,63;0,78;0,93)	0,111
	E4	(0,3;0,5;0,7)	75	(0,27;0,47;0,67)	0,111
	E5	(0,1;0,25;0,4)	50	(0,14;0,29;0,44)	0,074
	E6	(0,1;0,25;0,4)	50	(0,15;0,3;0,45)	0,074
	E7	(0,3;0,5;0,7)	50	(0,35;0,55;0,75)	0,074
	E8	(0,3;0,5;0,7)	75	(0,33;0,53;0,73)	0,111
	E9	(0,3;0,5;0,7)	50	(0,26;0,46;0,66)	0,074
	E10	(0,3;0,5;0,7)	75	(0,33;0,53;0,73)	0,111
	E11	(0,1;0,25;0,4)	0	(0;0;0)	0,000
	E12	(0,6;0,75;0,9)	50	(0,66;0,81;0,96)	0,074
	Final Measure to 2.1.2			Normal (0,48;0,02)	

According to Table 4, Expert 11 has not been coherent in their opinions, and so this was excluded from the risk aggregation ' Flood and storm risks'. S9 Fig shows the opinion distribution function adjusted for some experts regarding the language scale distribution functions.

Transitivity laws were applied to the pair-wise comparison opinions collected on risk discrimination, to check for consistency. From the scalar value retrieved in the additive transitivity law application according to Eq (15), the judgements of the experts are critically examined at the three hierarchy levels using Eq (16), and the *D* local value is determined using Eq (17).

Application of the model has shown that experts have a Confidence Level that did not require modification of the risk description, with a maximum of five incoherent experts on risk probability for Risks 3.2.1 and 3.2.2.

Table 5 shows a hierarchical risk discrimination aggregation on the Impact on Scope for technological risks, as a result of applying Eq (18). Once the data have been added, local *ORF* parameters on the Scope, Cost, and project Time are estimated according to Eq (19) as well as the global *ORF* using Eq (20) as shown in Table 6.

**Table 5:** *RDIS* hierarchy aggregation: Technological risks.

GROUP	DIS 1^st^ Level	SUB-GROUP	DIS 2^nd^ Level	RISK	DIS 3^rd^ Level	RDIS Aggregated
2.-	Normal (0,55;0,02)	2.1.	Beta General (3,97;3,33;0,42;0,58)	2.1.1.	Weibull (3,58;0,07;Shift(0,43))	Weibull (3,26;0,05;Shift(0,10))
				2.1.2.	Beta General (6,36;5,99;0,45;0,58)	Weibull (3,38;0,05;Shift(0,10))
				2.1.3.	Normal (0,36;0,02)	Weibull (3,33;0,04;Shift(0,07))
				2.1.4.	Weibull (4,30;0,06;Shift(0,49))	Beta General (6,02;6,29;0,10;0,20)
				2.1.5.	Weibull (4,00;0,06;Shift(0,45))	Weibull (3,57;0,05;Shift(0,10))
		2.2.	Beta General (4,69;3,99;0,39;0,55)	2.2.1.	Weibull (7,42;0,12;Shift(0,35))	Normal (0,12;0,01)
				2.2.2.	Normal (0,43;0,02)	Weibull (3,39;0,04;Shift(0,08))
				2.2.3.	Weibull (3,97;0,06;Shift(0,38))	Weibull (3,36;0,04;Shift(0,08))
				2.2.4.	Weibull (4,11;0,07;Shift(0,51))	Weibull (3,47;0,05;Shift(0,10))
				2.2.5.	Normal (0,52;0,04)	Weibull (3,44;0,05; Shift(0,10)
				2.2.6.	Weibull (7,06;0,14;Shift(0,40))	Normal (0,14;0,01)

**Table 6:** Estimate ORF: Plant location.

RISK	ORFS	ORFC	ORFT	ORF
2.1.1.	Invgauss (7,31;87,71;Shift(1,74))	Lognorm (5,54;1,53;Shift(1,26))	Gamma (6,71;0,55;Shift(2,50))	Invgauss (58,24;244,67;Shift(4,69))
2.1.2.	Gamma (5,53;1,43;Shift(6,95))	Gamma (5,21;1,94;Shift(9,55))	Invgauss (12,87;127,43;Shift(4,65))	Invgauss (245,34;913,71;Shift(29,32))
2.1.3.	Gamma (5,49;2,60;Shift(12,30))	Gamma (4,63;3,03;Shift(13,83))	Gamma (5,99;1,04;Shift(4,99))	Invgauss (544,80;2028,92;Shift(64,71))
2.1.4.	Gamma (5,77;0,68;Shift(3,44))	Gamma (4,67;1,02;Shift(4,90))	Gamma (5,18;1,23;Shift(5,82))	Gamma (2,38;36,58;Shift(39,44))
2.1.5.	Gamma (5,17;1,43;Shift(7,56))	Gamma (5,08;1,43;Shift(7,40))	Gamma (5,24;1,97;Shift(9,96))	Invgauss (321,99;1245,72;Shift(49,12))

### Risk ranking

Assessing differential risk impact on the Scope, Cost, or project Time is an important contribution, since previous studies have only evaluated the impact of risk on the general project framework. As a result of applying the model to the case study, we have obtained a risk ranking that allows us to plan the general risk response to the Scope, Cost, and Time.

## Results and discussion

A comprehensive risk analysis method such as the one presented in this paper is critical for adequate risk response planning. To obtain a risk ranking, working directly with distribution functions, the Mode value is chosen as the most frequent value, which is equivalent to the centroid method employed in the classic fuzzy methodology.

Based on the ranking results within the general framework project (S10 Fig) it will be necessary to focus the efforts of the project management team towards the risks ‘Bank financing’, ‘Changes in energy prices’, ‘Specific legislation changes’, ‘The change in energy policy’, ‘Delays in obtaining the construction license’. Of the ten most influential risks in the general framework of the project, the majority are those due to delays. Therefore, in order to ensure the future success of the project, the risk management team should focus its efforts on controlling project deadlines, whilst not forgetting bank financing and possible changes in the electricity market price.

In order to compare the operation of the proposed model, the risk ranking has been determined using the classical fuzzy methodology, resulting in the ranking shown in S11 Fig.

As can be seen, the first 4 risks of the ranking agree, although not in terms of their order of influence over the general framework of the project. The variations in ranking positions imply the need for a different response when planning against their impact on the success of the project. According to the classic methodology, ‘Bank financing’, ‘Changes in energy prices’ and ‘Time delays risks’ are still fundamental, but ‘Agreement with the land owners’ would go from being a priority risk to being in sixth place in the proposed method, and would go from being a secondary risk to being ranked eleventh in accordance with the classical methodology. These differences are presented in a generalized manner throughout the risk ranking.

The proposed method allows for a specific analysis of the influence of the risks identified in the construction projects of photovoltaic plants with respect to their influence on scope, costs, and deadlines.

Analyzing the importance of the risks in terms of project Scope, the main risks worthy of attention are ‘Specific legislation changes’, ‘Bank financing’, ‘Changes in energy prices’, ‘The change in energy policy’, ‘Construction delays of the power connection infrastructure’. Among the first ten risks, in this case, the economic risks have greater influence than the risks due to delays, and the risk of theft appears in seventh place in the ranking.

As far as costs are concerned, the most important risks are ‘Changes in energy prices’, ‘Bank financing’, ‘Agreement cost with land-owners’, ‘Theft’, ‘Specific legislation changes’. As with the case of scope, the most important risks in terms of costs are, logically, the economic risks. In this case, the risk of theft is ranked in fourth place and risks due to delays have less influence on the costs of the project.

Finally, the greatest risks for project Time are ‘Bank financing’, ‘Delays in obtaining the construction license’, `Delays in obtaining approval of the environmental impact’, ‘The change in energy policy’, ‘Construction delays of the power connection infrastructure’. Logically, from the point of view of the project term, the most important risks are those related to delays.

## Conclusions

This paper presents a new methodology for risk analysis that is both qualitative and quantitative, based on probabilistic analysis, detailed risk definitions, and expert opinion. Unlike the classic fuzzy sets methodology that works with triangular numbers that are eventually simplified, this work uses whole probabilistic functions and Monte Carlo simulation to obtain more complete results for the same database, thus allowing for a better analysis. The main points to note are as follows:

- This new approach introduces a new parameter in risk analysis, known as ‘Confidence Level’. This new parameter analyses the linguistic aspects of expert opinion, varying the answers according to their coherence and weighting their judgements regarding impact and probability.
- From the ‘Confidence Level’ parameter we can evaluate the quality of the risk descriptions, since if at least half of the experts do not show coherence this indicates that the risk description is incorrect and therefore must be redefined, and experts should again express their opinions on all the levels associated with the redefined risk.
- The proposed methodology examines the application of transitivity laws, which not only serve to determine the consistency of the experts’ opinions regarding discrimination between risks, but also weights the consistency of their judgments.
- To obtain the ranking that allows for planning the risk response, the distribution function Mode value is used, which is equivalent to the centroid method used in the classical methodology.

A case study of a real renewable energy project—a 250 MW Photovoltaic Plant located in Spain—was used to test the model. When compared with the classical fuzzy sets, the PFSAHP results in a more efficient method for both qualitative and quantitative risk analyses based on linguistic assessments in the early stages of large-scale renewable energy construction projects.

- The new application is smooth and agile application, with a rapid and thorough methodology that could be readily transferred to the analysis of other construction projects for obtaining a risk ranking from which to plan a risk response strategy.
- From the point of view of the general framework of the project, the most important risks are obtaining bank financing, changes in energy prices in the electricity market, changes in sector-specific legislation, changes in the energy policy introduced by the government of the country, and delays in obtaining the works license.
- A specific analysis of the influence of risks from the point of view of scope, cost and time frame has been carried out, allowing us to draw the following conclusions:
  - Scope: Economic risks have a greater influence than risks from delays. The risk of theft must be taken into account when having greater influence on the scope.
  - Cost: Since economic risks are those that have the strongest influence on cost, the risk of theft is a significant risk.
  - Time: Along with bank financing and changes in sector-specific legislation, the most important risks are those related to delays.

## Supporting information

S1 Fig Proposed PFSAHP methodology. (TIF)

S2 Fig RI linguistic terms and fuzzy numbers. (TIF)

S3 Fig RP linguistic terms and fuzzy numbers. (TIF)

S4 Fig RD linguistic terms and fuzzy numbers. (TIF)

S5 Fig RI tuning fuzzy numbers. (TIF)

S6 Fig ORF. (TIF)

S7 Fig Risk ranking. (TIF)

S8 Fig Risk breakdown structure. (TIF)

S9 Fig Risk impact matt. (TIF)

S10 Fig Mode ORF risks ranking. (TIF)

S11 Fig Fuzzy classical ORF risks ranking. (TIF)

S1 File Renamed_b81c5. (XLSM)

S2 File EXPERT_QUESTIONNAIRES ENGLISH. (XLSM)

S3 File Survey responses. (RAR)

## References

[1] Perry JG, Hayes RW, 1985 Risk and its management in construction projects Proceeding of Institution Civil Engineers, 499–521. 10.1680/iicep.1985.859

[2] Chapman CB, Ward SC, 1997 Project risk management: Processes, Techniques and Insights. Wiley.

[3] Aven T, 2016 Risk assessment and risk management: review of recent advances on their foundation. European Journal of Operational Research, 116(2), 235–248. 10.1016/j.ejor.2015.12.023

[4] Serrano L, Muñoz JI, 2016. Risk identification in large photovoltaic plants’ construction projects. AEIPRO, 20th International Congress on Project Management and Engineering, Cartagena, 13-15th July 2016, 1786–1798.

[5] Zadeh LA, 1965 Fuzzy sets. Information Control 8, 338–353.

[6] Li Y, Wang X, 2016 Risk assessment for public–private partnership projects: using a fuzzy analytic hierarchical process method and expert opinion in China. Journal of Risk Research, 1–22, Published online 20 Dec 2016. 10.1080/13669877.2016.1264451

[7] Valipour A, Yahaya N, Md Noor N, Kildienė S, Sarvari H, Mardani A, 2015 A fuzzy analytic network process method for risk prioritization in freeway PPP projects: an Iranian case study. Journal of Civil Engineering and Management 21, 933–947. 10.3846/13923730.2015.1051104

[8] Ferreira AC, Franklin C M, 2007 Fuzzy inference to risk assessment on nuclear engineering systems. Applied Soft Computing 7, 17–28. 10.1016/j.asoc.2005.06.002

[9] Xiaojun W, Hing KC, Dong L, 2015 A case study of an integrated fuzzy methodology for green product development. European Journal of Operational Research 241, 212–223. 10.1016/j.ejor.2014.08.007

[10] Yao-Chen K, Shih-Tong L, 2013 Using fuzzy multiple criteria decision making approach to enhance risk assessment for metropolitan construction projects. International Journal of Project Management 31, 602–614. 10.1016/j.ijproman.2012.10.003

[11] Nieto-Morote A, Ruz-Vila F, 2011 A fuzzy approach to construction project risk assessment. International Journal of Project Management 29, 220–231. 10.1016/j.ijproman.2010.02.002

[12] Gatzert N, Vogl N, 2016 Evaluating investments in renewable energy under policy risks. Energy Policy 95, 238–252. 10.1016/j.enpol.2016.04.027

[13] Shafiee M, 2015 A fuzzy analytic network process model to mitigate the risks associated with offshore wind farms. Expert Systems with Applications 42, 2143–2152. 10.1016/j.eswa.2014.10.019

[14] Chen G, 2015. Risk analysis and evaluation of wind electric farm construction. Proceedings of the AASRI International Conference on Industrial Electronics and Applications (IEA 2015), 539–543.

[15] Liu Y, Dai XT, Meng QZ, 2015. Research on Risk Assessment of Photovoltaic Building Project by Triangular Fuzzy Number Method. Proceedings of the 2015 International Conference on Education Reform and Modern Management (ERMM 2015), 354–358.

[16] Jiuping X, Huan Z, Ziqiang Z, Shiyong W, Manbin S, 2012 Discrete time–cost–environment trade-off problem for large-scale construction systems with multiple modes under fuzzy uncertainty and its application to Jinping-II Hydroelectric Project. International Journal of Project Management 30, 950–966. 10.1016/j.ijproman.2012.01.019

[17] Yan F, Xu KL, Yao XW, and Li Y, 2016 Fuzzy Bayesian network-bow-tie analysis of gas leakage during biomass gasification. PloS One. 11(7). 10.1371/journal.pone.0160045 27463975 PMC4963130

[18] Yu L, Li YP, Huang GH, 2016 A fuzzy-stochastic simulation-optimization model for planning electric power systems with considering peak-electricity demand: A case study of Qingdao, China. Energy 98, 190–203. 10.1016/j.energy.2016.01.021

[19] Wu CB, Huang GH, Li W, Zhen JL, Ji L, 2015 An inexact fixed-mix fuzzy-stochastic programming model for heat supply management in wind power heating system under uncertainty. Journal of Cleaner Production 112, 1717–1728. 10.1016/j.jclepro.2015.04.061

[20] Içen D, Demirhan H, 2016 Error measures for fuzzy linear regression: Monte Carlo simulation approach. Applied Soft Computing 46, 104–114. 10.1016/j.asoc.2016.04.013

[21] Nguyen HY, Dawal SZ, Nukman Y, Aoyama H, Case K, 2015 An Integrated Approach of Fuzzy Linguistic Preference Based AHP and Fuzzy COPRAS for Machine Tool Evaluation. PloS One 10(9): e0133599 10.1371/journal.pone.0133599 26368541 PMC4569346

[22] Yan F, Xu KL, Cui ZK, and Yao XW, 2017 An improved layer of protection analysis based on a cloud model: Methodology and case study. Journal of Loss Prevention in the Process Industries 48, 41–47. 10.1016/j.jlp.2017.04.006

[23] Yang Q, Du P-A, Wang Y, Liang B, 2017 A rough set approach for determining weights of decision makers in group decision making. PloS One 12(2): e0172679 10.1371/journal.pone.0172679 28234974 PMC5325315

[24] Lyons T, Skitmore M, 2004 Project risk management in the Queensland engineering construction industry: a survey. International Journal of Project Management 22, 51–61. 10.1016/S0263-7863(03)00005-X

[25] Xu Z, Zhang X, 2013 Hesitant fuzzy multi-attribute decision making based on TOPSIS with incomplete weight information. Knowledge-Based Systems 52, 53–64. 10.1016/j.knosys.2013.05.011

[26] Zeng J, A M and Smith NJ, 2007 Application of a fuzzy based decision making methodology to construction project risk assessment. International Journal of Project Management 25, 6 589–600. 10.1016/j.ijproman.2007.02.006

[27] Zhao HR, Li NN, 2015 Risk Evaluation of a UHV Power Transmission Construction Project Based on a Cloud Model and FCE Method for Sustainability. Sustainability 7, 2885–2914.

[28] Hsu CF, Li RK, Kang HY, Lee AHI, 2014 A Systematic Evaluation Model for Solar Cell Technologies. Mathematical Problems in Engineering. 10.1155/2014/542351

[29] Kang MS, Chen CS, Ke YL, Lee AHI, Ku TT, Kang HY, 2012 Applications of FANP and BOCR in Renewable Energy-Study on the Choice of the Sites for Wind Farms. IEEE Transactions on Industry Applications 49, 982–989. 10.1109/TIA.2013.2243395

[30] Bin Z, Zeshui X, Ren Z, Mei H, 2015 Generalized analytic network process. European journal of Operational Research 244, 277–288. 10.1016/j.ejor.2015.01.011

[31] Hu J, Zhou E, 2011 Engineering risk management planning in energy performance contracting in China. Engineering and Risk Management 1, 195–205. 10.1016/j.sepro.2011.08.032

[32] Yim R, Castaneda J, Doolen T, Tumer I, Malak R, 2015 A study of the impact of project classification on project risk indicators. International Journal of Project Management 33, 863–876. 10.1016/j.ijproman.2014.10.005

[33] Ahmed A, Kayis B, Amornsawadwatana S, 2007 A review of techniques for risk management in projects. Benchmarking 14, 22–36. 10.1108/14635770710730919

[34] Chapman R, 1998 The effectiveness of working group risk identification and assessment techniques. International Journal of Project Management 16, 333–343. 10.1016/S0263-7863(98)00015-5

[35] Rezaei J, Ortt JR, 2013 Multi-criteria supplier segmentation using a fuzzy preference relations based AHP. European Journal of Operational Research 225, 75–84. 10.1016/j.ejor.2012.09.037

[36] Kaplan S, Garrick BJ (1981). On the Quantitative Definition of Risk. Risk Analysis, I(I), 11–27. 10.1111/0272-4332.215153 11798118

[37] Cervone HF, 2006 Project Risk management. OCLC Systems & Services 22, 256–262.

[38] Kaufmann A, Gupta MM, 1991 Introduction to fuzzy arithmetic: Theory and Application. Van Nostrand Reinhold, New York.

[39] Dubois D, Prade H, 1978 Operations on fuzzy numbers. International Journal of Systems Science 9, 613–626.

[40] Kannan D, de Sousa Jabbour ABL, Jabbour CJC, 2014 Selecting green suppliers based on GSCM practices: Using fuzzy TOPSIS applied to a Brazilian electronics company. European Journal of Operational Research 233, 432–447. 10.1016/j.ejor.2013.07.023

[41] Tanino T, 1988 Fuzzy preference relations in group decision making Non-Conventional Preference Relations in Decision Making. Springer-Verlag, Berlin, 54–71.

[42] Herrera F, Herrera-Viedma E, Martínez L, 2000 A fusion approach for managing multi-granularity linguistic term sets in decision making. Fuzzy Sets and Systems 114, 43–58. 10.1016/S0165-0114(98)00093-1

[43] Herrera F, Herrera-Viedma E, 1997. Choice functions and mechanisms for linguistic preference relations. Dept. Computer Sciences and A.I. Granada University, Technical Report, DECSAI-97134.

[44] Taylan O, Bafail AO, Reda MSA, Kabli MR, 2014 Construction projects selection and risk assessment by fuzzy AHP and fuzzy TOPSIS methodologies. Applied Soft Computing 17, 105–116. 10.1016/j.asoc.2014.01.003

[45] Yan F, Xu KL, 2018 A set pair analysis based layer of protection analysis and its application in quantitative risk assessment. Journal of Loss Prevention in the Process Industries 55, 313–319. 10.1016/j.jlp.2018.07.007

[46] Lazim A, Norsyahida Z, 2015 Integration of fuzzy AHP and interval type-2 fuzzy DEMATEL: An application to human resource management. Expert Systems with Applications 42, 9, 4397–4409. 10.1016/j.eswa.2015.01.021

[47] Aragonés-Beltrán P, Chaparro-González F, Pastor-Ferrando JP, Rodríguez-Pozo F, 2009 An ANP-based approach for the selection of photovoltaic solar power plant investment projects. Renewable and Sustainable Energy Reviews 14, 249–264. 10.1016/j.rser.2009.07.012

[48] Athanasios K, Read G, Ioannou A, 2016 Application of multi-criteria decision-making to risk prioritization in tidal energy developments. International Journal of Sustainable Energy 35, 59–74. 10.1080/14786451.2014.880438

[49] Kucukali S, 2011 Risk assessment of river-type hydropower plants using fuzzy logic approach. Energy Policy 39 (2011) 6683–6688. 10.1016/j.enpol.2011.06.067

[50] Cucurachi S, Borgonovo E, Heijungs R, 2016 A Protocol for the Global Sensitivity Analysis of Impact Assessment Models in Life Cycle Assessment. Risk Analysis 36, 357–377. 10.1111/risa.12443 26595377

