# Supplementary figures and images for: Monte Carlo approach to fuzzy AHP risk analysis in renewable energy construction projects
Source: PLoS One. 2019 Jun 13;14(6):e0215943. doi: 10.1371/journal.pone.0215943 (PMC6563964; doi:10.1371/journal.pone.0215943)

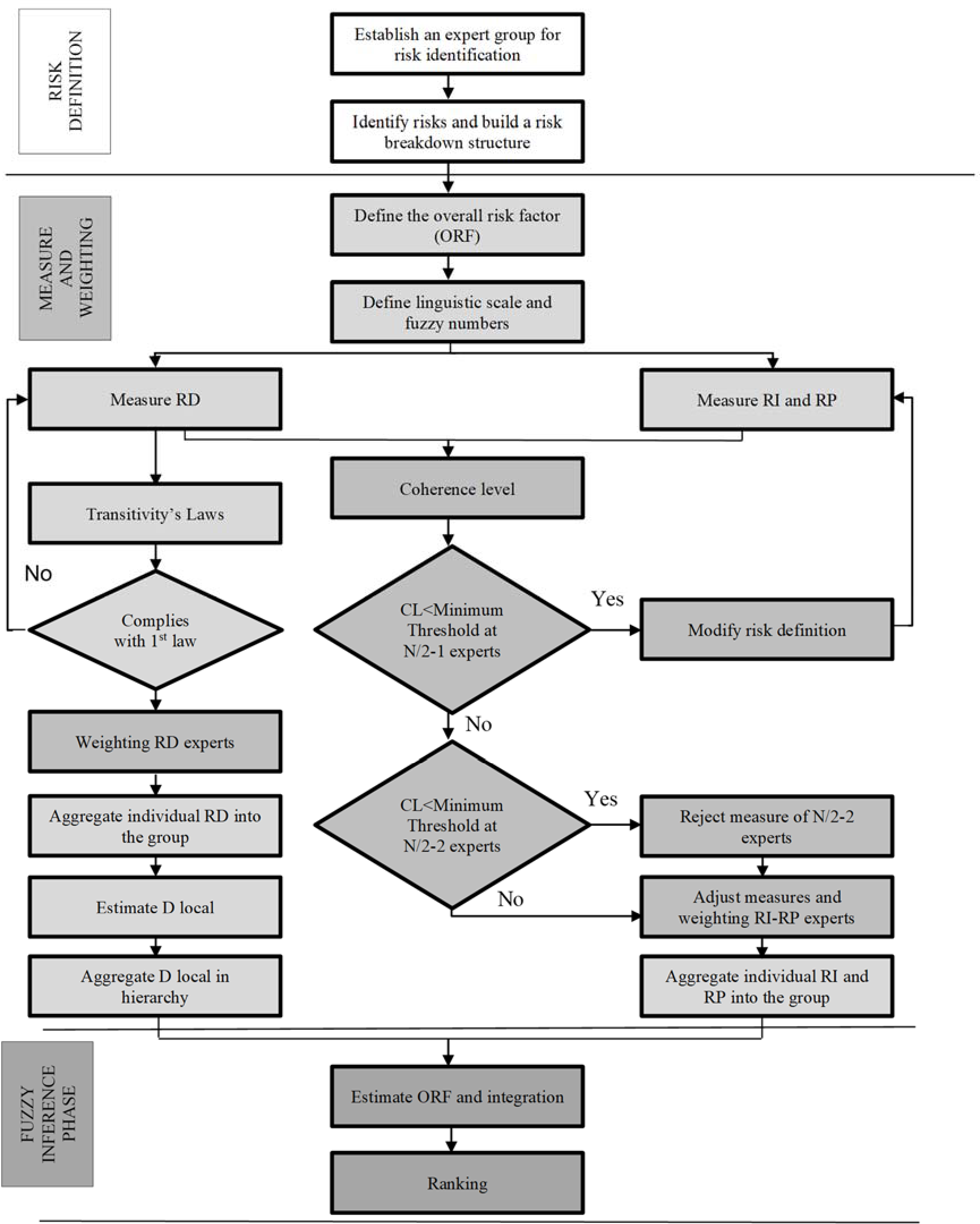

Supplement: S1 Fig — (TIF) [file pone.0215943.s001.tif]

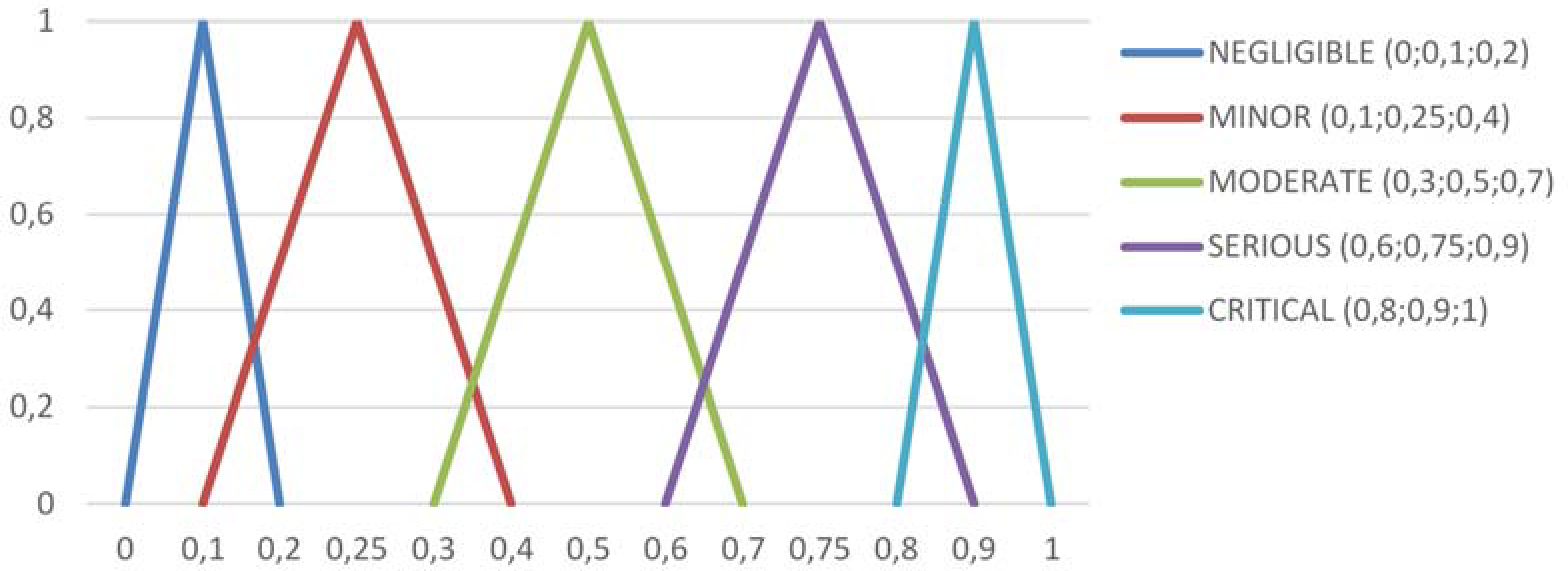

Supplement: S2 Fig — (TIF) [file pone.0215943.s002.tif]

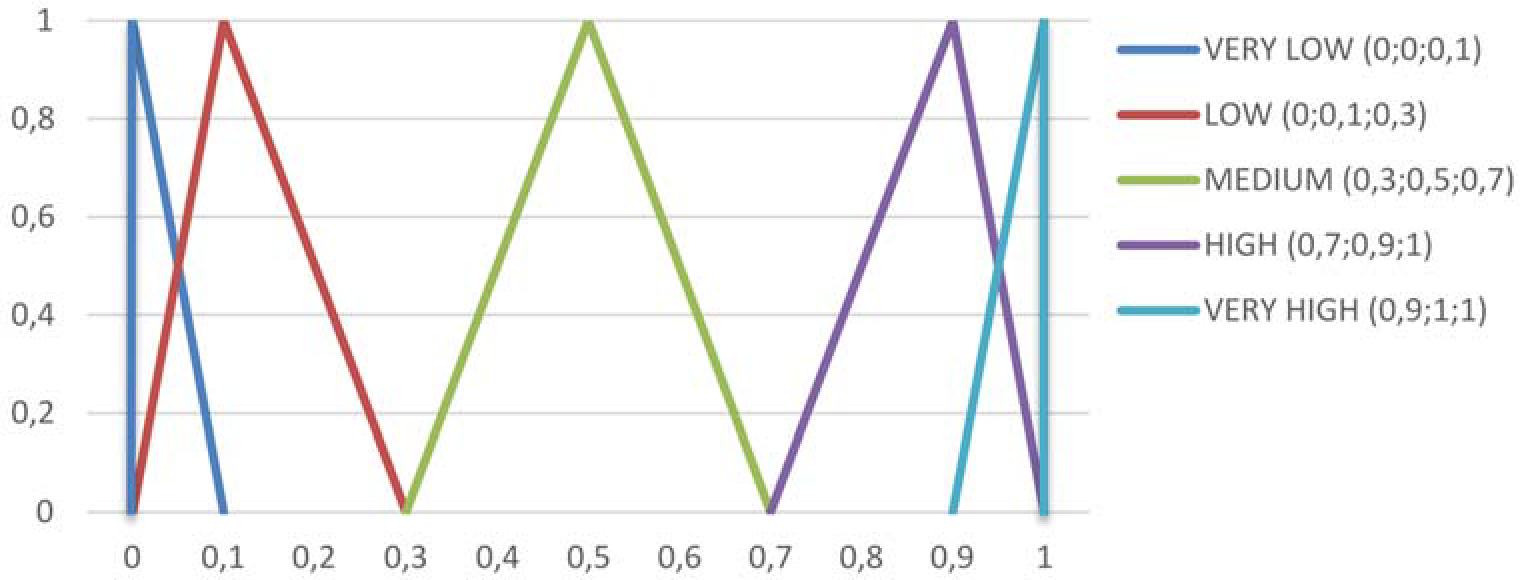

Supplement: S3 Fig — (TIF) [file pone.0215943.s003.tif]

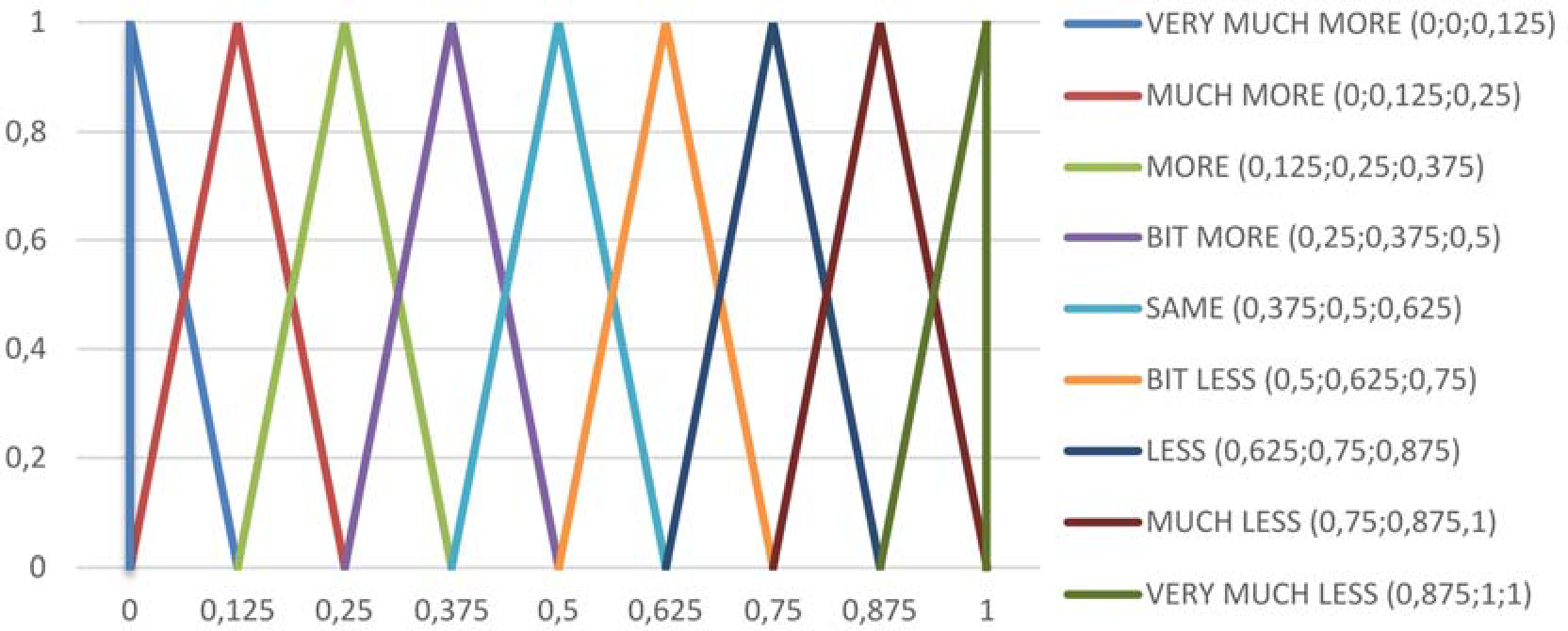

Supplement: S4 Fig — (TIF) [file pone.0215943.s004.tif]

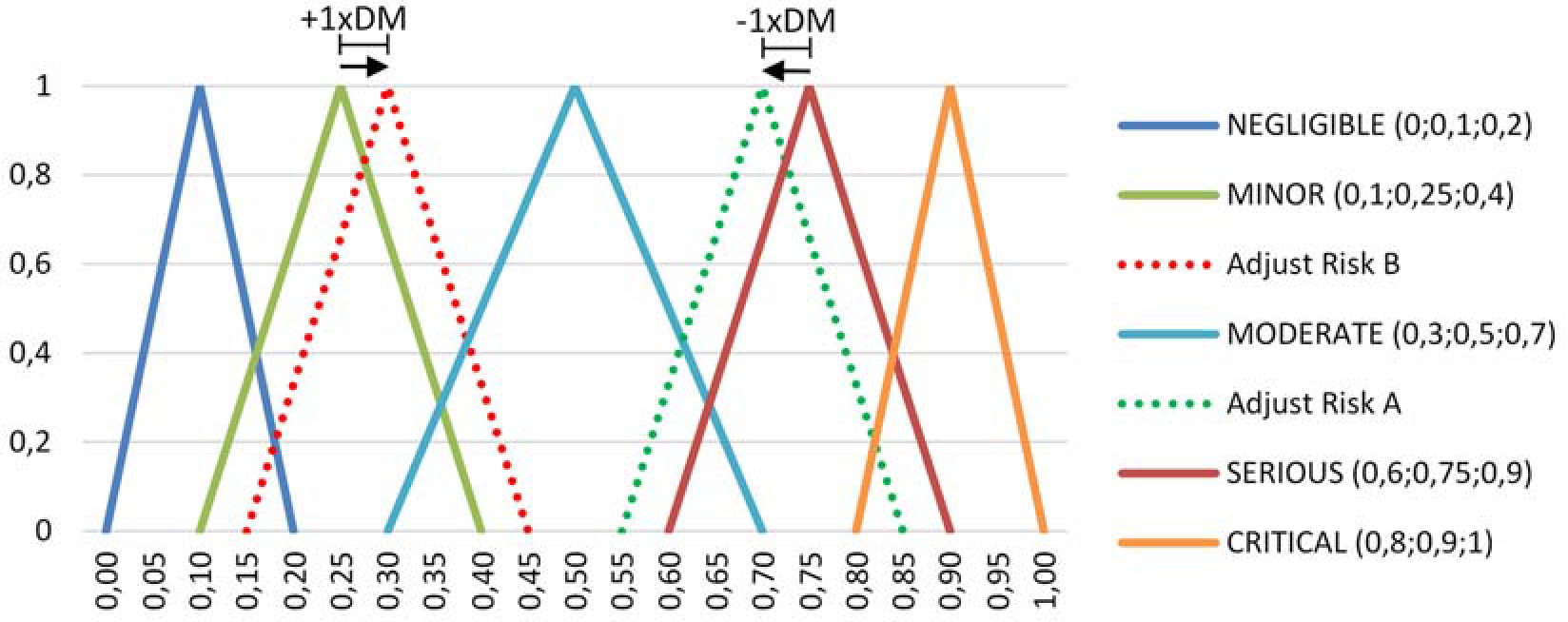

Supplement: S5 Fig — (TIF) [file pone.0215943.s005.tif]

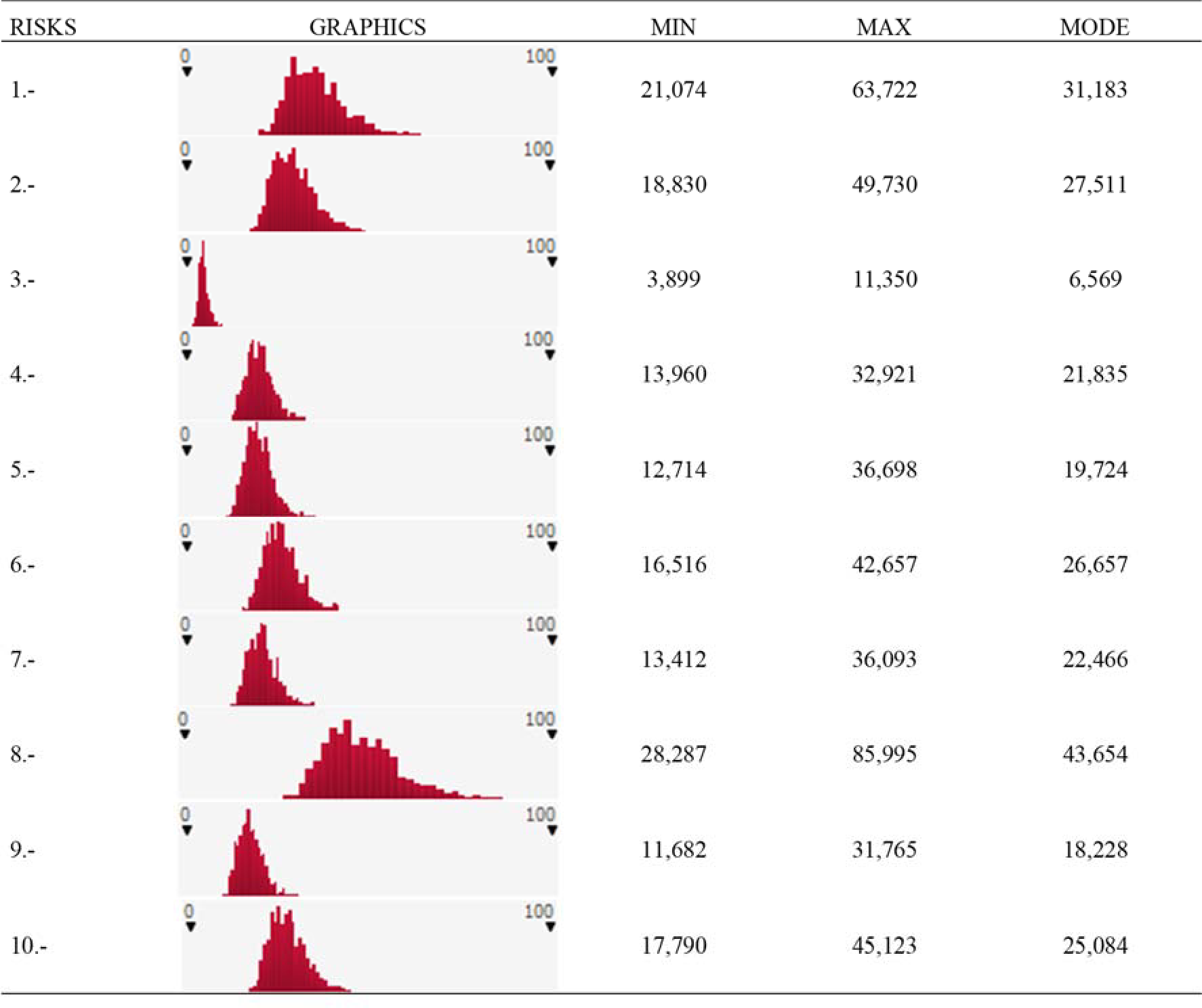

Supplement: S6 Fig — (TIF) [file pone.0215943.s006.tif]

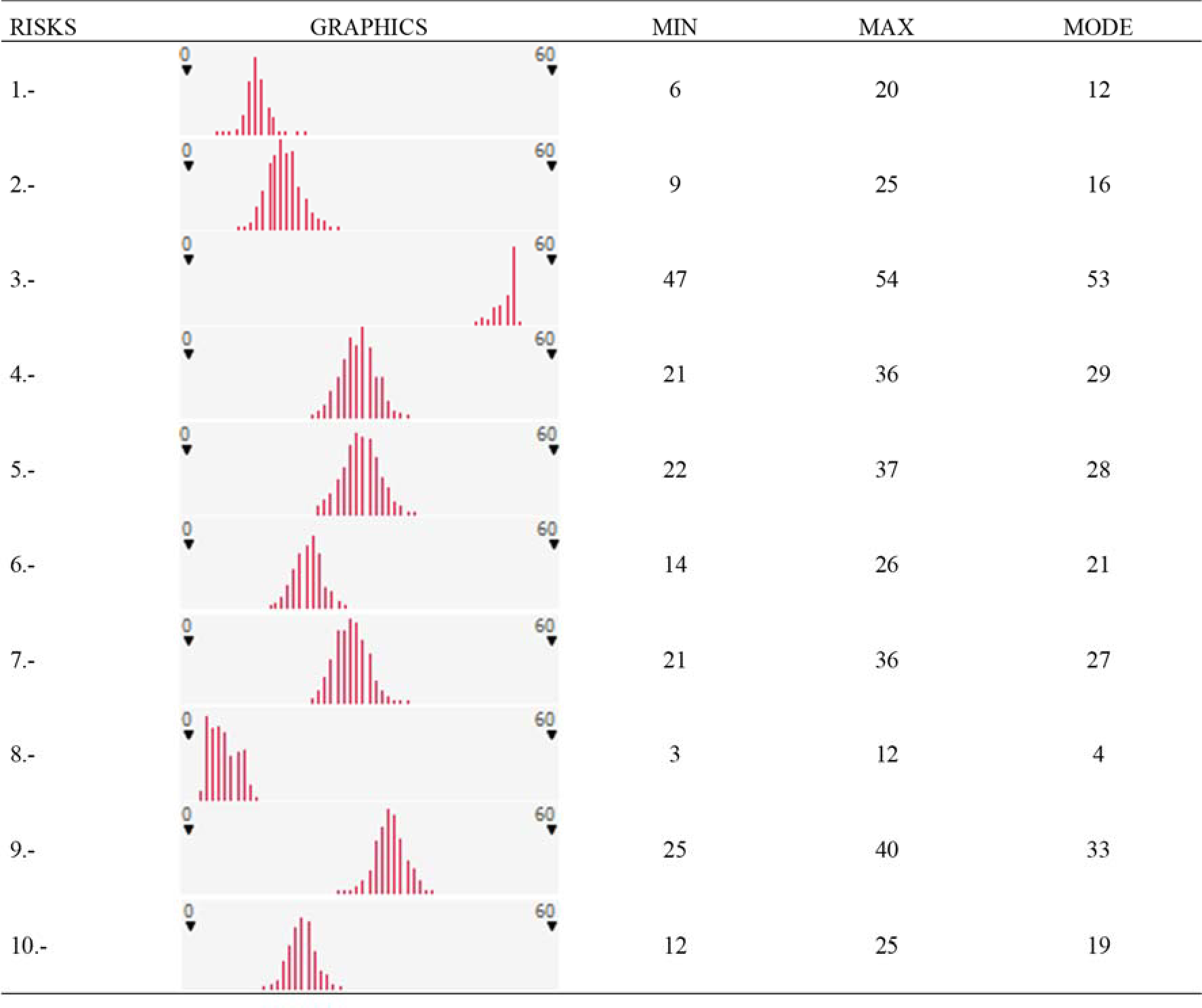

Supplement: S7 Fig — (TIF) [file pone.0215943.s007.tif]

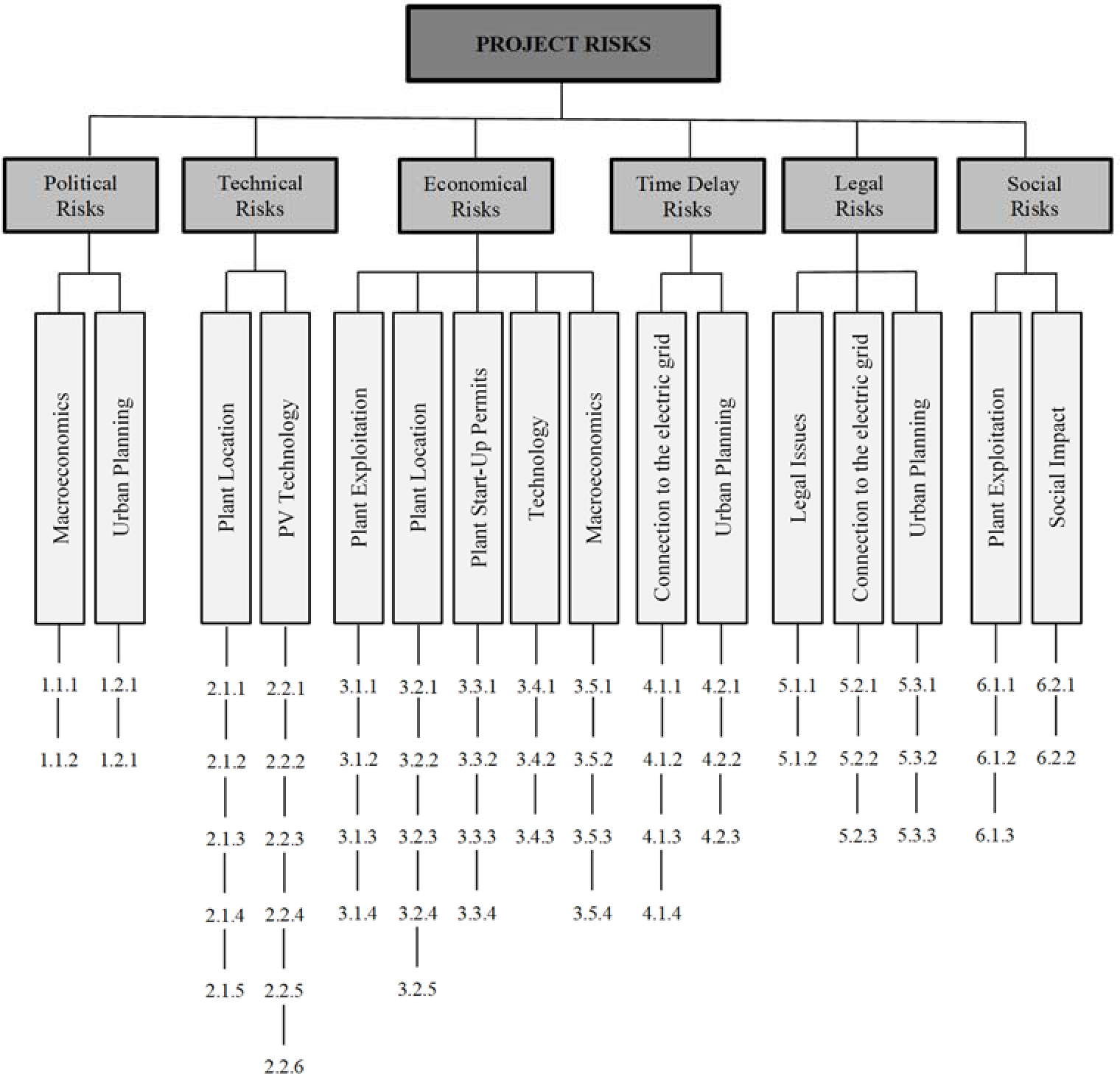

Supplement: S8 Fig — (TIF) [file pone.0215943.s008.tif]

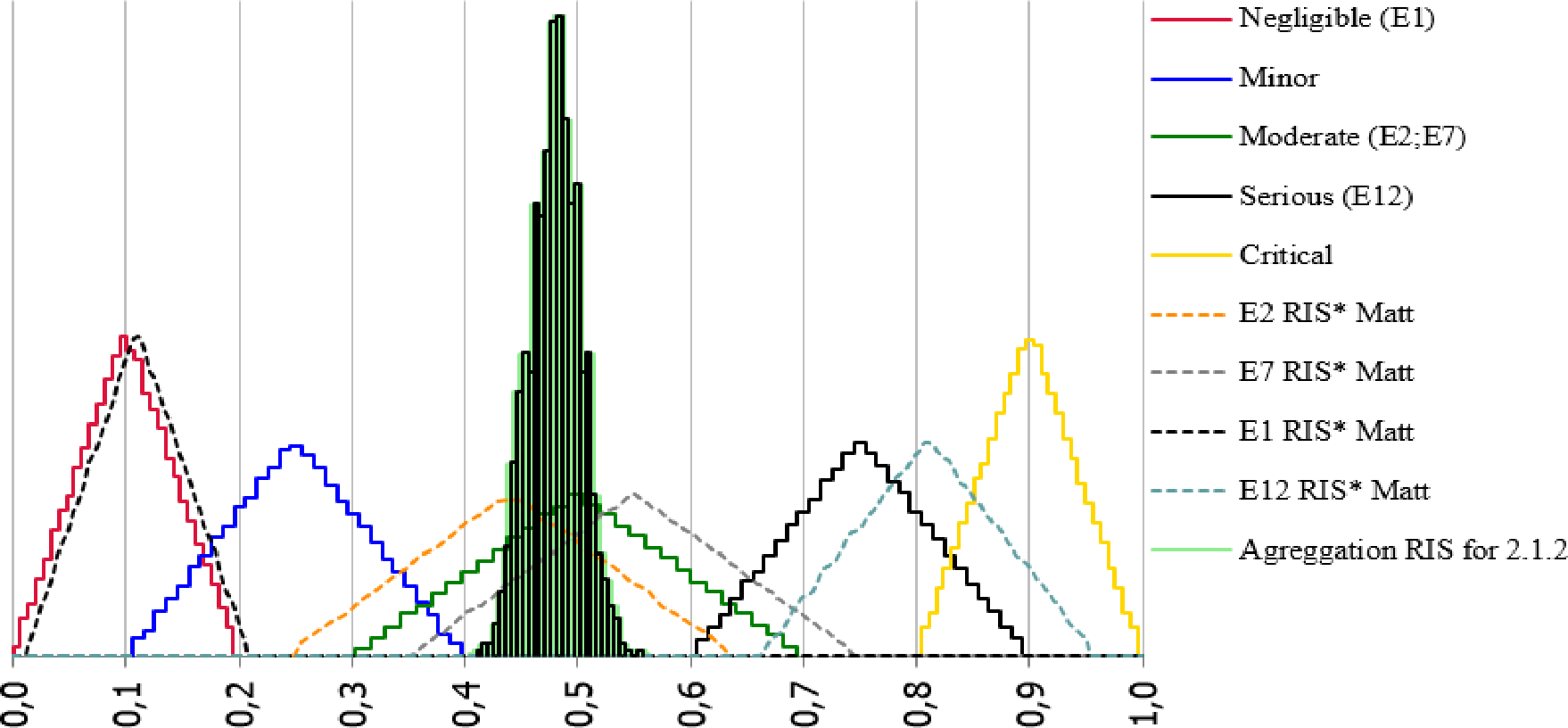

Supplement: S9 Fig — (TIF) [file pone.0215943.s009.tif]

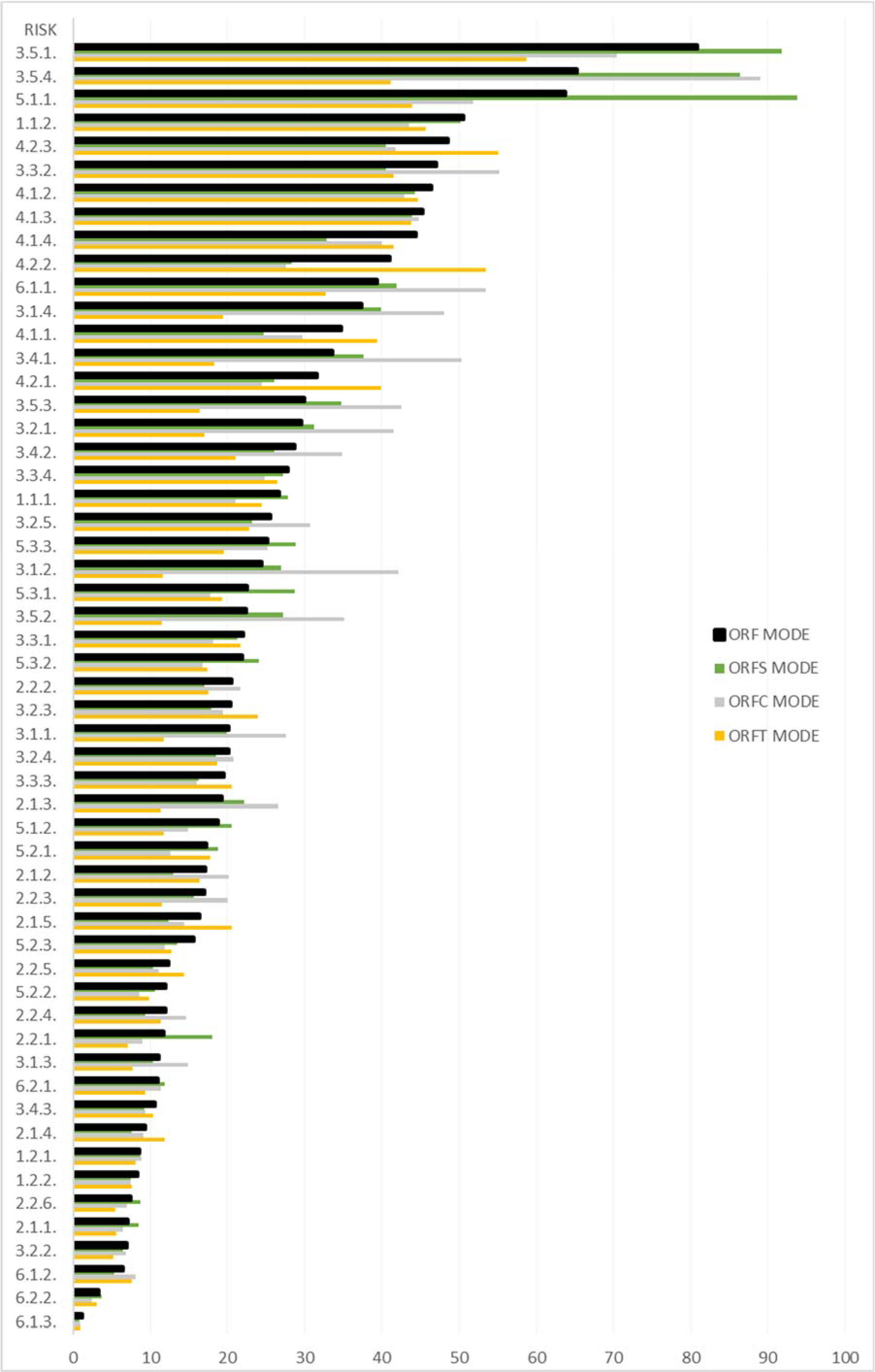

Supplement: S10 Fig — (TIF) [file pone.0215943.s010.tif]

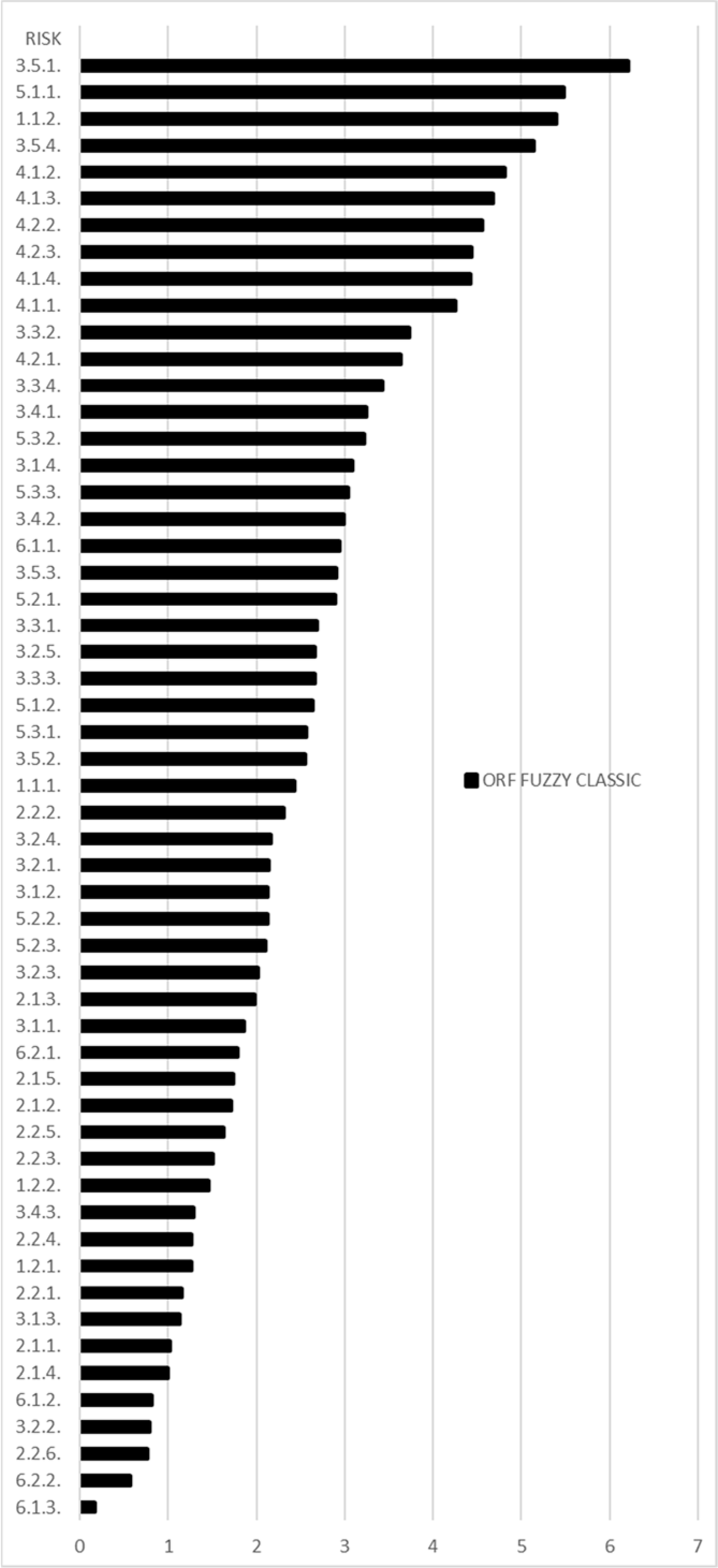

Supplement: S11 Fig — (TIF) [file pone.0215943.s011.tif]
